# Comparing functional and quality of life outcomes in transcatheter aortic valve implantation and surgical aortic valve replacement for aortic stenosis: a systematic review and meta-analysis

**DOI:** 10.1186/s12872-023-03445-y

**Published:** 2023-10-25

**Authors:** Michael Duffy, Amy Lynch, Catriona Reddin, Conor Judge, Martin O’Donnell, Robert Murphy

**Affiliations:** 1 HRB-Clinical Research Facility, University of Galway, Galway, Ireland; 2 Galway University Hospital, Newcastle Road, Galway, Ireland; 3 Welcome Trust – HRB, Irish Clinical Academic Training, Galway, Ireland; 4 Division of Neurology, Sunnybrook Health Sciences Centre, Toronto, ON Canada; 5 Division of Neurology, Department of Medicine, University of Toronto, Toronto, ON Canada

**Keywords:** Aortic stenosis, Transcatheter Aortic Valve Implantation (TAVI), Surgical Aortic Valve Replacement (SAVR), Quality of life

## Abstract

**Background:**

To compare functional and health related quality of life outcomes post-transcatheter aortic valve implantation (TAVI) and surgical aortic valve replacement (SAVR) in patients with critical aortic stenosis (AS) across low to high-risk surgical candidates. These patient-centred factors will be compared between both groups in the short to medium term time frames and will aid in shared decision making between patients and healthcare workers.

**Materials and methods:**

We conducted a systematic review and meta-analysis of randomised controlled trials which compared TAVI with SAVR and reported on quality of life (QoL) and functional scores.

The scores used were the Kansas City Cardiomyopathy Questionnaire (KCCQ), Euroqol-5DL (EQ5DL), the short form-36/12 (SF-36/12) and the NYHA.

**Results:**

We identified eight trials with a total of 8898 participants. Both groups showed improvements from baseline at one month. At one month there was a statistically significant difference in standardised mean difference (SMD) in favour of TAVI for EQ5DL (SMD 0.37, 95% CI 0.26,0.49), KCCQ (SMD 0.53,95% CI 0.48, 0.58), SF physical summary (SMD 0.55, 95% CI 0.31 – 0.78) and SF mental summary (SMD 0.34, 95% CI 0.27 – 0.40). At one year there was no statistically significant difference between any of these QoL metrics. For NYHA, no significant difference in odds ratio of class III/IV was observed at one month between TAVI and SAVR (OR 0.94, 95% CI 0.83, 1.07), however, TAVI was associated with reduced odds ratio of NYHA class I/II at one year (OR 0.87, 95% CI 0.78, 0.98).

**Conclusion:**

Both groups were associated with improvements in QoL and functional outcomes with TAVI reporting more significant improvements in QoL at one-month post-procedures. No significant improvements between groups were seen at one year. This is the largest meta-analysis comparing post-operative health-related quality of life outcomes post SAVR and TAVI and has major implications in shared decision making for the treatment of aortic stenosis.

**Supplementary Information:**

The online version contains supplementary material available at 10.1186/s12872-023-03445-y.

## Key points

**Aim:**

To compare functional and health related quality of life outcomes across all surgical risk groups post-transcatheter aortic valve implantation (TAVI) and surgical aortic valve replacement (SAVR) in patients with critical aortic stenosis (AS).

**Findings:**

Both groups were associated with improvements in QoL and functional outcomes with TAVI reporting more significant improvements in QoL at one-month post-procedures. No significant improvements between groups were seen at one year.

**Message:**

Greater short-term improvements in quality of life/functional outcome measures seen in the TAVI group are likely due to its less invasive nature and this should be taken into consideration in patient-centred decision-making.

**Supplementary Information:**

The online version contains supplementary material available at 10.1186/s12872-023-03445-y.

## Introduction

Transcatheter aortic valve implantation (TAVI) can be considered as a treatment option over surgical aortic valve repair (SAVR) across all patients from high-risk to low risk patient groups [1]. Previous meta-analyses have found that compared with surgical aortic valve replacement (SAVR), TAVI has similar or reduced mortality rates, with a suggestion of a lower risk of fatal stroke or disabling stroke with TAVI in more recent trials and in studies which had higher rates of transfemoral route access [2–4]. Choice of intervention; TAVI, SAVR or conservative management, may be influenced by a variety of elements including anatomical factors such as suitability for transfemoral access, patient preference and goals for quality of life [5]. Health-related quality of life (HRQOL) outcomes and functional outcomes following intervention provide additional information to guide shared decision making.

A prior meta-analysis in 2018 explored the health-related quality of life outcomes and functional outcomes post-TAVI and SAVR [6]. Ando et al. found statistically significant differences in HRQOL scores in trans-femoral TAVI compared to SAVR for both heart failure specific and generic health assessment tools at one-month post-operatively [6]. Since this meta-analysis a number of additional trials have been published (e.g., PARTNER 3, Evolut Surgical Replacement and Transcatheter Aortic Valve Implantation in Low Risk Patients, UK TAVI Trial) [7–9], as well as secondary analyses of previous RCTs which reported on functional outcomes [10, 11]. In light of these studies additional analysis is warranted to see if statistically significant differences in TAVI over SAVR are limited to just high risk patients and to explore if differences are apparent at longer term follow up.

The objective of this meta-analysis was to complete an updated meta-analysis of health-related quality of life outcomes and functional outcomes in patients undergoing intervention for critical aortic stenosis. We aim to extend previous analyses by exploring both short term (30 days) and medium term (1 year) outcomes in a number of additional studies and incorporating additional functional outcomes. We aim to answer if improvements in health-related quality of life outcomes are restricted to mainly high risk surgical candidates and this will offer additional insights for clinicians when engaging in shared decision making with patients.

## Methods

We performed a systematic review and meta-analysis, adhering to the Cochrane Collaboration Guidelines and the Preferred Reporting Items for Systematic Reviews and Meta-Analyses (PRISMA) Guidelines [12, 13]. The meta-analysis was registered with the International Prospective Register of Systematic Reviews (PROSPERO identifier: CRD42022343243 ((https://www.crd.york.ac.uk/prospero/display_record.php?ID=CRD42022343243).

**Fig. 1:**
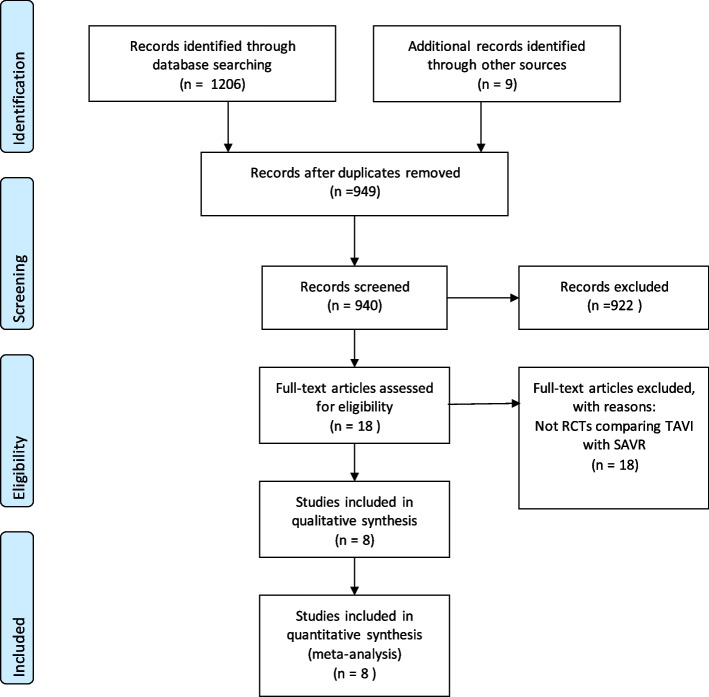
Flow diagram

Figure 1 shows a flow diagram representing the selection process for all included papers.

### Data sources and search strategy

To reduce research waste [14], we extracted data from a recent meta-analysis of quality of life and functional outcomes post TAVI or SAVR [6]. This approach of completing a new cumulative meta-analysis allowed it to be designed and interpreted in the context of relevant systematic reviews which aids in the accumulation of results. We considered this meta-analysis of sufficiently high quality to avoid repeating the primary search. We limited our search to dates not included in this review (26^th^ October 2017 onwards).

We systematically searched PubMed and Embase databases from the 26^th^ of October 2017 to 29^th^ June 2022. Two reviewers (MD and RM) screened titles and abstracts using the Rayyan web application [15]. The reference list of included studies were also reviewed. Full texts of remaining articles were independently assessed by two reviewers (MD and RM), with eligibility based on pre-determined criteria. Disagreements were resolved by consensus, where a resolution was not reached by discussion, a consensus was reached through a third reviewer (MOD).

### Eligibility criteria

Studies were considered eligible if they were: [1] randomised controlled trials; [2] comparing TAVI versus SAVR across all surgical risk categories; [3] reported functional outcomes using a clinical scoring systems or health-related quality of life scale, such as Euroqol-5 (EQ5D), the Kansas City Cardiomyopathy Questionnaire (KCCQ), the Short form-12/36 (SF-12/36), the New York Heart Association Classification score (NYHA); and [4] reported short-term and one year outcomes. Studies were excluded if they were non-randomised controlled trials, single-armed papers assessing TAVI or SAVR or studies that did not report on health-related outcome measures.

### Data extraction

Data was extracted independently by two authors (MD and RM) using a standardized pre-determined data collection form. Original publications and follow-up studies up were assessed. For each study, we extracted the title, year of publication, follow-up duration, valve type, intervention and control, percentage of women, participant numbers, functional outcomes or health-related quality of life scores (EQ5DL, KCCQ, SF12/16 and NYHA). We extracted data at one month and one year, except for EVOLUT trial which reported data at six weeks instead of one month for early functional and quality of life outcomes [7]. Data was compared for inconsistencies and merged into a final dataset.

### Outcomes

The primary outcome was a change in functional scores. The outcomes gathered were the EQ5D, KCCQ, SF-12/36, the NYHA score. The EQ5D and SF-12/36 were used as generic health-related quality of life scores offering versatile quantification of overall post-operative health status by assessing quality of life from a biopsychosocial perspective. The SF12/36 report a physical summary and mental summary score. The NYHA and KCCQ offered a tailored analysis of disease-specific limitations and improvements in participants.

### Data synthesis and analysis

Data for EQ5D and KCCQ were reported as standardised means difference and standard deviation (SD). The summary scores of SF-12 and SF-36 describe the same construct and were meta-analysed together, with subgroup analysis conducted by the individual scores [16]. The proportion of patients with mild heart failure symptoms (NYHA I/II) versus more severe heart failure symptoms (NYHA III/IV) at follow up were compared. NYHA was reported as a dichotomised odds ratio with 95% confidence intervals (CI).

An inverse variance statistical model and random effects model was used to synthesise continuous data and calculate the mean difference and 95% CI. The variability across studies due to heterogeneity was estimated with the I^2^ statistic. In sensitivity analysis we explored effect-modification for year of study publication and mean age of study participants. Statistical analysis was performed using the Metafor package on R Statistical Software [17].

### Bias and quality assessment

The risk of bias was evaluated using the Cochrane collaboration Risk of Bias 2 tool [18]. The domains assessed were selective outcome reporting, incomplete outcome data, blinding of outcome assessment, blinding of participants and personnel, sequence generation: allocation concealment, and other issues. The risk of bias was categorised into low, high or uncertain risk.

## Results

We identified eight randomised clinical trials, published from 2011–2022, that reported on various quality of life and functional scores with a total of 8898 participants (Fig. 1). The Staccato randomised clinical trial from 2012 did not report on quality of life or functional scores so was excluded [19]. The characteristics of included trials are outlined in Table 1. Of the eight trials, three were in low surgical risk groups [7, 8, 20], three in intermediate risk [9]and two in high risk [21, 22]. Sample sizes ranged from 278 to 2053 participants. The mean age of trial participants was 79.5 years old with 42.1% of trial participants female.

**Table 1:** Baseline characteristics of selected studies

Study	Year	No. Treated with TAVI	No. Treated with SAVR	STS Risk Score	Percentage Women	Average Age	Type of Valve
Partner 1	2011	344	313	11.8	42.7%	84.1	Sapien Heart
CoreValueUS	2014	390	357	7.4	46.7%	83.4	CoreValve self-expanding prosthesis
Notion	2015	139	135	3	46.7%	79.1	CoreValve self-expanding prosthesis
Partner 2A	2016	994	944	5.8	45.4	81.6	Sapien XT valve system
Surtavi	2017	864	796	4.5	43.2	79.8	CoreValve Bioprostheiss, Evolut R bioprosthesis
Evolut	2019	725	678	2	34.9%	74	CoreValve, EvolutR, Evolut PRO
Partner 3	2019	496	454	1.9	30.7%	73.4	Balloon- expandable SAPIEN 3 system
UK TAVI	2022	450	419	2.7	46.4%	81	Any

### EQ5DL

**Fig. 2:**
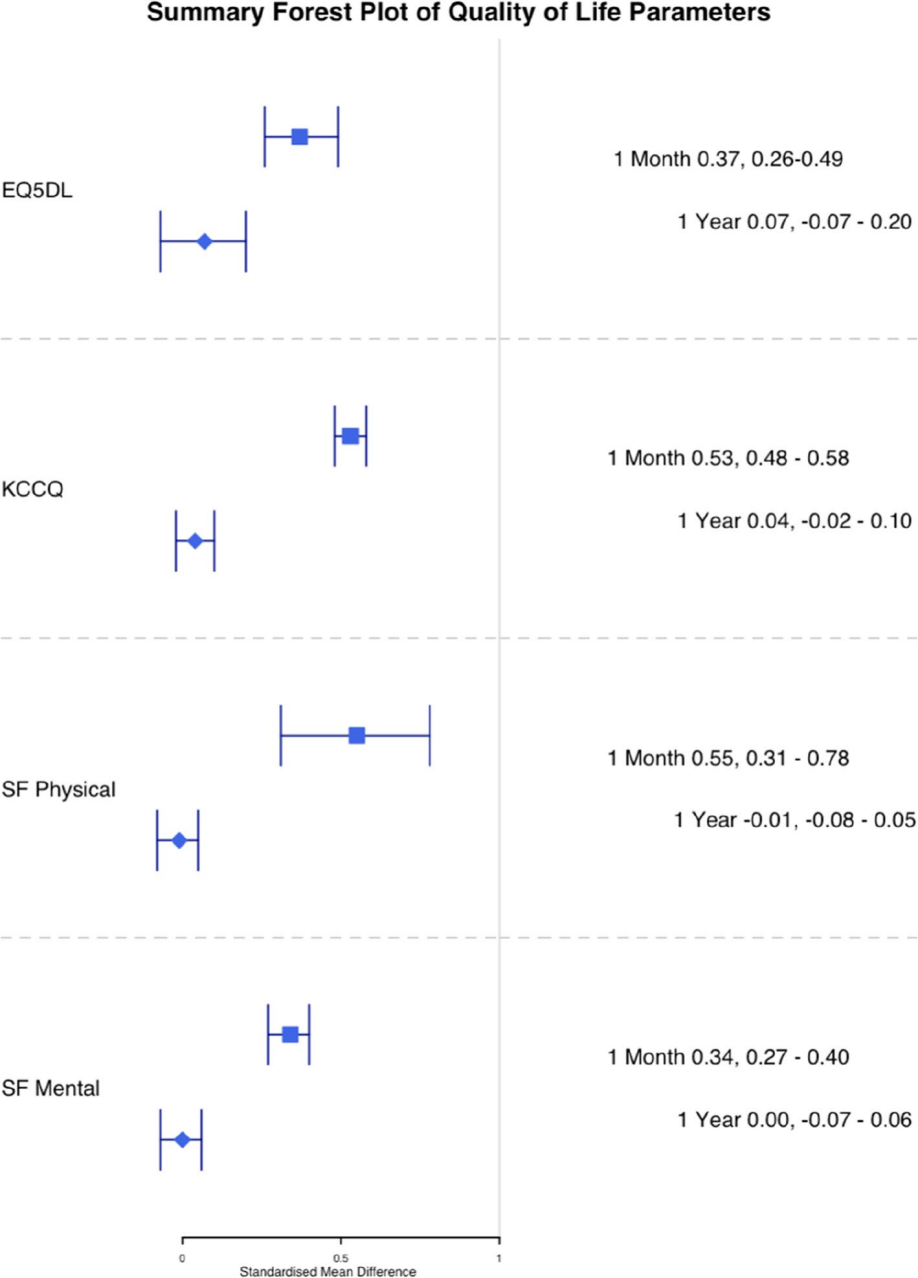
Summary forest plot of quality of life parameters. Figure 2 shows a summary forest plot of EQ5DL, KCCQ, SF Physical and SF Mental scores comparing mean differences between TAVI and SAVR at one month and one year. Abbreviations: EQ5D (Euroqol 5 Dimensions score), KCCQ (Kansas city cardiomyopathy questionnaire), SF (Short Form), TAVI (Transcatheter aortic valve Implantation), SAVR (Surgical Aortic Valve Replacement), SMD (Standardised mean difference)

**Fig. 3:**
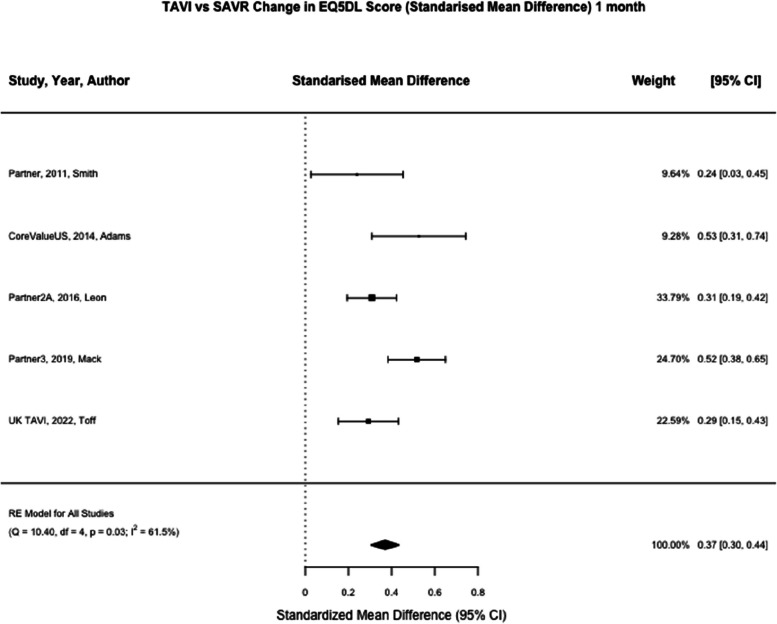
TAVI vs SAVR change in EQ5DL score (Standarised Mean Difference) 1 month. Figure 3 shows a forest plot comparing the standardised mean difference of EQ5DL scores between TAVI and SAVR at one month. Abbreviations: EQ5DL (Euroqol-5) TAVI (Transcatheter aortic valve implantation), SAVR (Surgical Aortic valve replacement), SMD (Standardised Mean Difference)

**Fig. 4:**
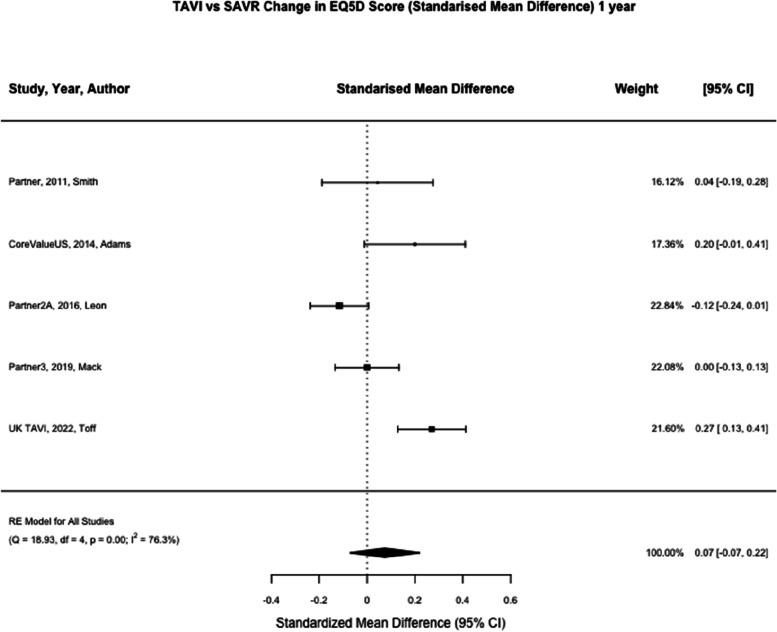
TAVI vs SAVR change in EQ5D score (Standarised Mean Difference) 1 year. Figure 4 shows a forest plot comparing the standardised mean difference of EQ5DL scores between TAVI and SAVR at one year. Abbreviations: EQ5DL (Euroqol-5) TAVI (Transcatheter aortic valve implantation), SAVR (Surgical Aortic valve replacement), SMD (Standardised Mean Difference)

EQ5DL was reported at one month in five studies (*n* = 3621) and one year in five studies (*n* = 3344) [8, 9, 21–23]. At one month, TAVI was associated with a significant reduction in EQ5DL score compared to SAVR (SMD 0.37; 95% CI, 0.26–0.49, *p* < 0.01). At one year, TAVI was not associated with a significant reduction in EQ5DL compared to SAVR (SMD 0.07; 95% CI, -0.07–0.20, *p* = 0.35) (Figs. 2, 3 and 4). Meta-regression at one month by mean age of trial participants (*p* = 0.81) or year of study completion (0.09) was not significant nor at one year for mean age (*p* = 0.67) or year of study completion (*p* = 0.53).

### KCCQ

**Fig. 5:**
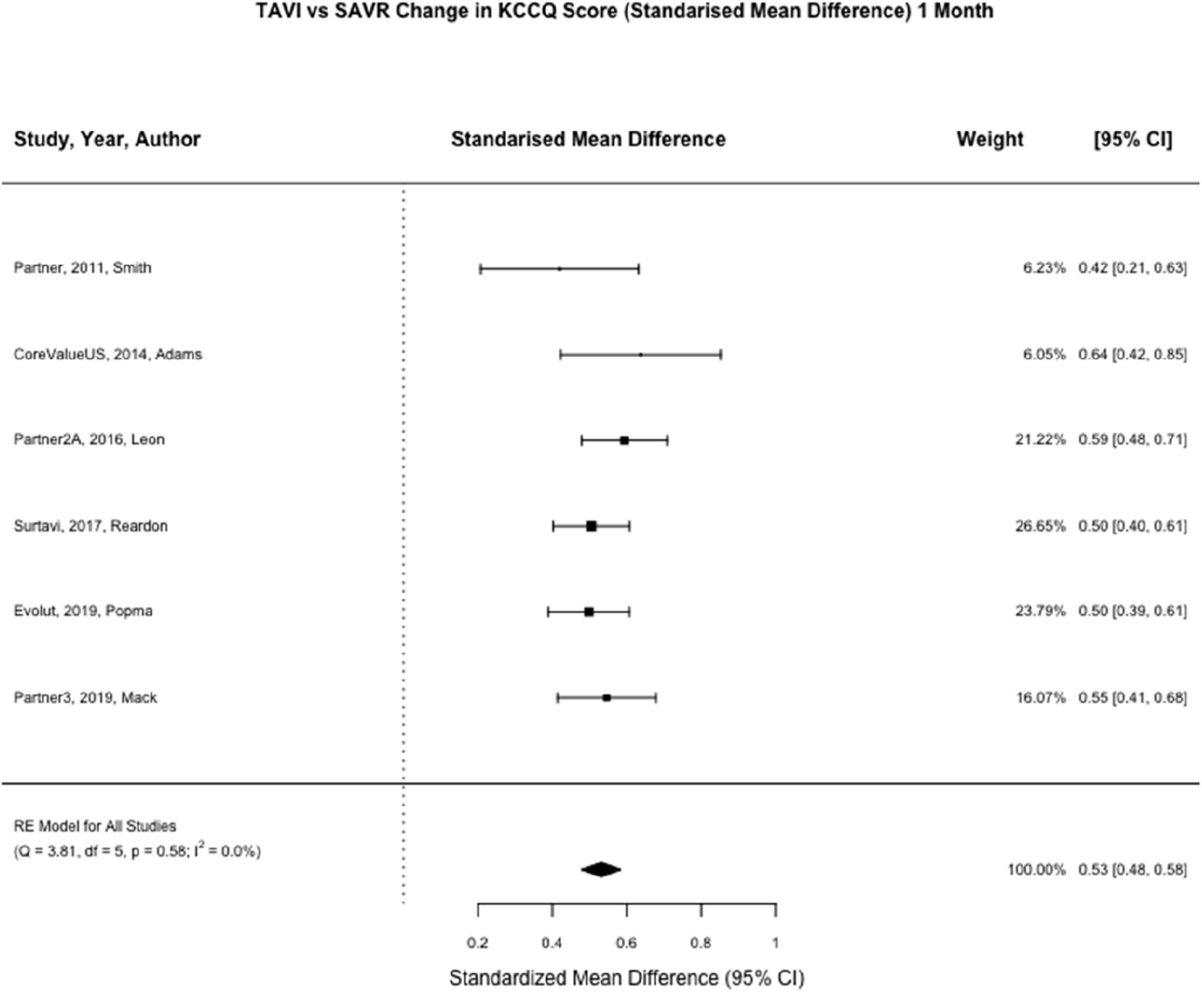
TAVI vs SAVR change in KCCQ score (Standarised Mean Difference) 1 month. Figure 5 shows a forest plot comparing the standardised mean difference of KCCQ scores between TAVI and SAVR at one month. Abbreviations: KCCQ (Kansas City Cardiomyopathy Questionnaire) TAVI (Transcatheter aortic valve implantation), SAVR (Surgical Aortic valve replacement), SMD (Standardised Mean Difference)

**Fig. 6:**
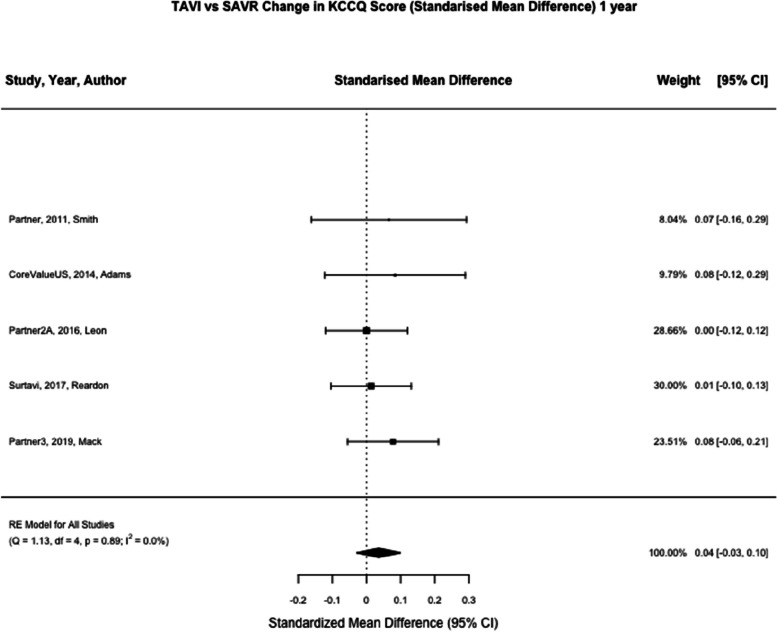
TAVI vs SAVR change in KCCQ score (Standarised Mean Difference) 1 year. Figure 6 shows a forest plot comparing the standardised mean difference of KCCQ scores between TAVI and SAVR at one year. Abbreviations: KCCQ (Kansas City Cardiomyopathy Questionnaire) TAVI (Transcatheter aortic valve implantation), SAVR (Surgical Aortic valve replacement), SMD (Standardised Mean Difference)

KCCQ was reported at one month in six studies (*n* = 5731) and one year in six studies (*n* = 4515) [7, 8, 21–24]. At one month, TAVI was associated with a significant reduction in KCCQ score compared to SAVR (SMD 0.53; 95% CI, 0.48 to 0.58, *p* < 0.01) (Fig. 5). At one year, TAVI was not associated with a significant reduction in KCCQ compared to SAVR (SMD 0.04; 95% CI, -0.02–0.10, *p* = 0.21) (Fig. 6).

### SF12/36:

**Fig. 7:**
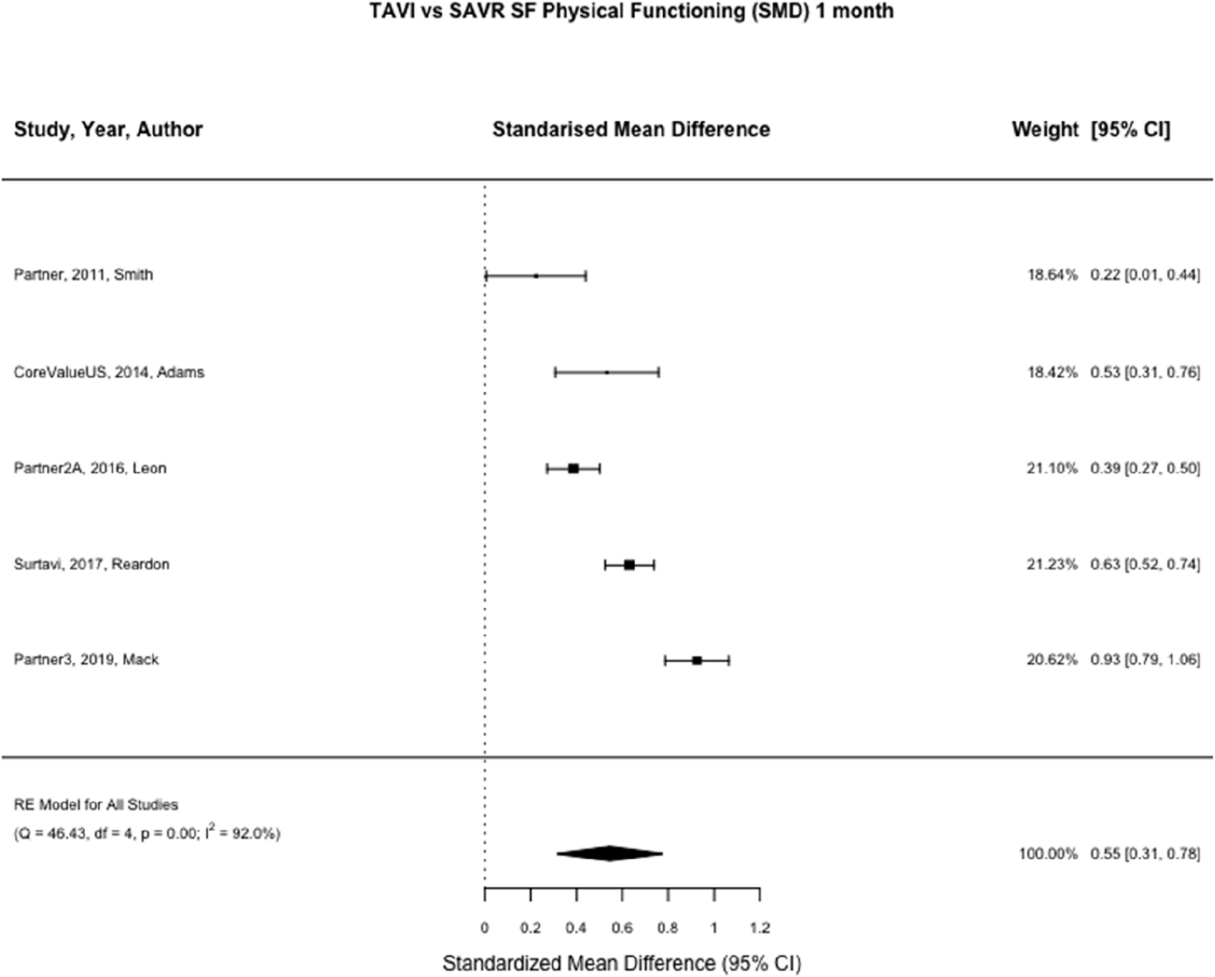
TAVI vs SAVR SF physical functioning (SMD) 1 month. Figure 7 shows a forest plot comparing the standardised mean difference of SF scores between TAVI and SAVR at one month. Abbreviations: SF Physical (Short Form Physical) TAVI (Transcatheter aortic valve implantation), SAVR (Surgical Aortic valve replacement), SMD (Standardised Mean Difference)

**Fig. 8:**
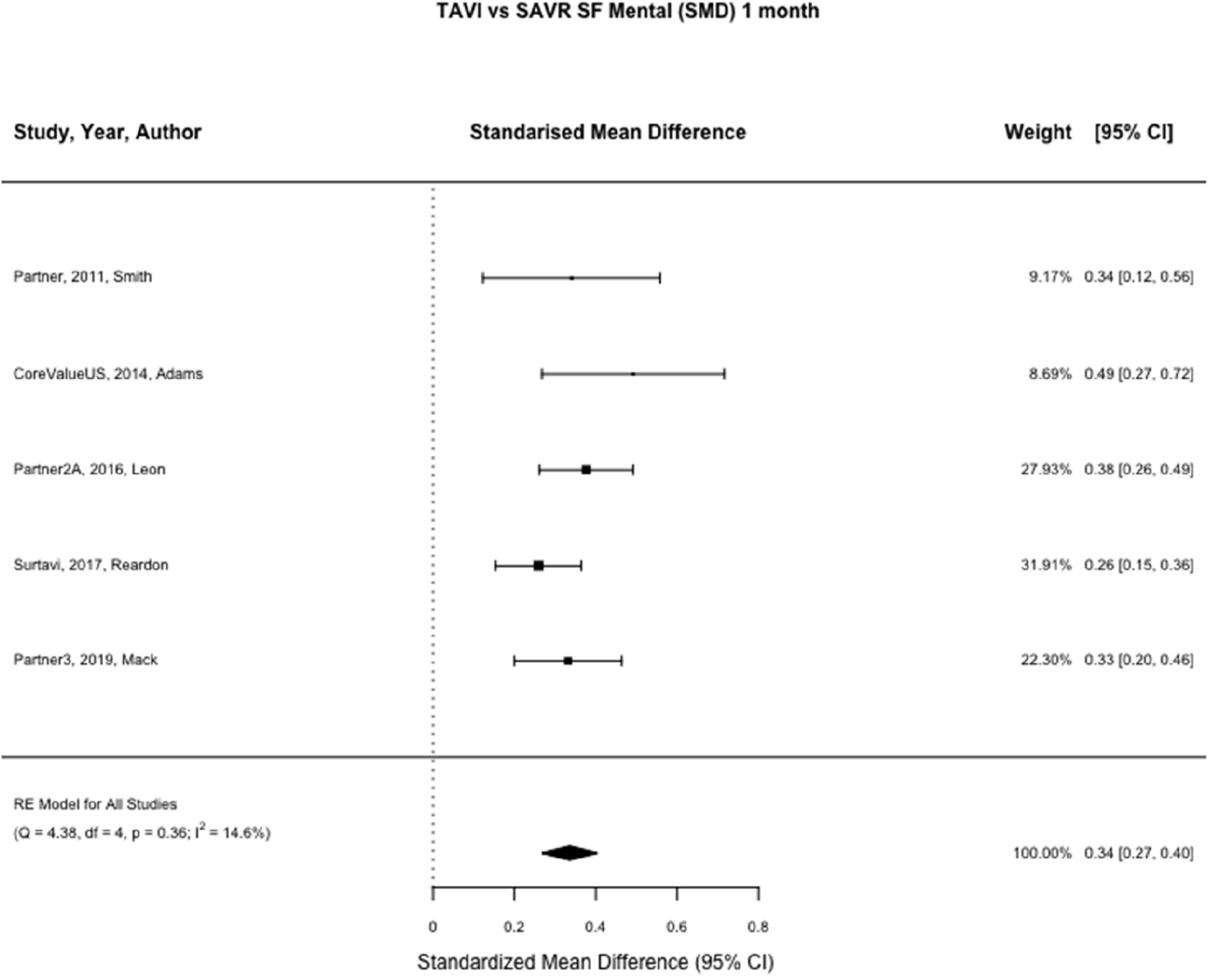
TAVI vs SAVR SF mental (SMD) 1 month. Figure 8 shows a forest plot comparing the standardised mean difference of SF scores between TAVI and SAVR at one month. Abbreviations: SF Mental (Short Form Mental) TAVI (Transcatheter aortic valve implantation), SAVR (Surgical Aortic valve replacement), SMD (Standardised Mean Difference

**Fig. 9:**
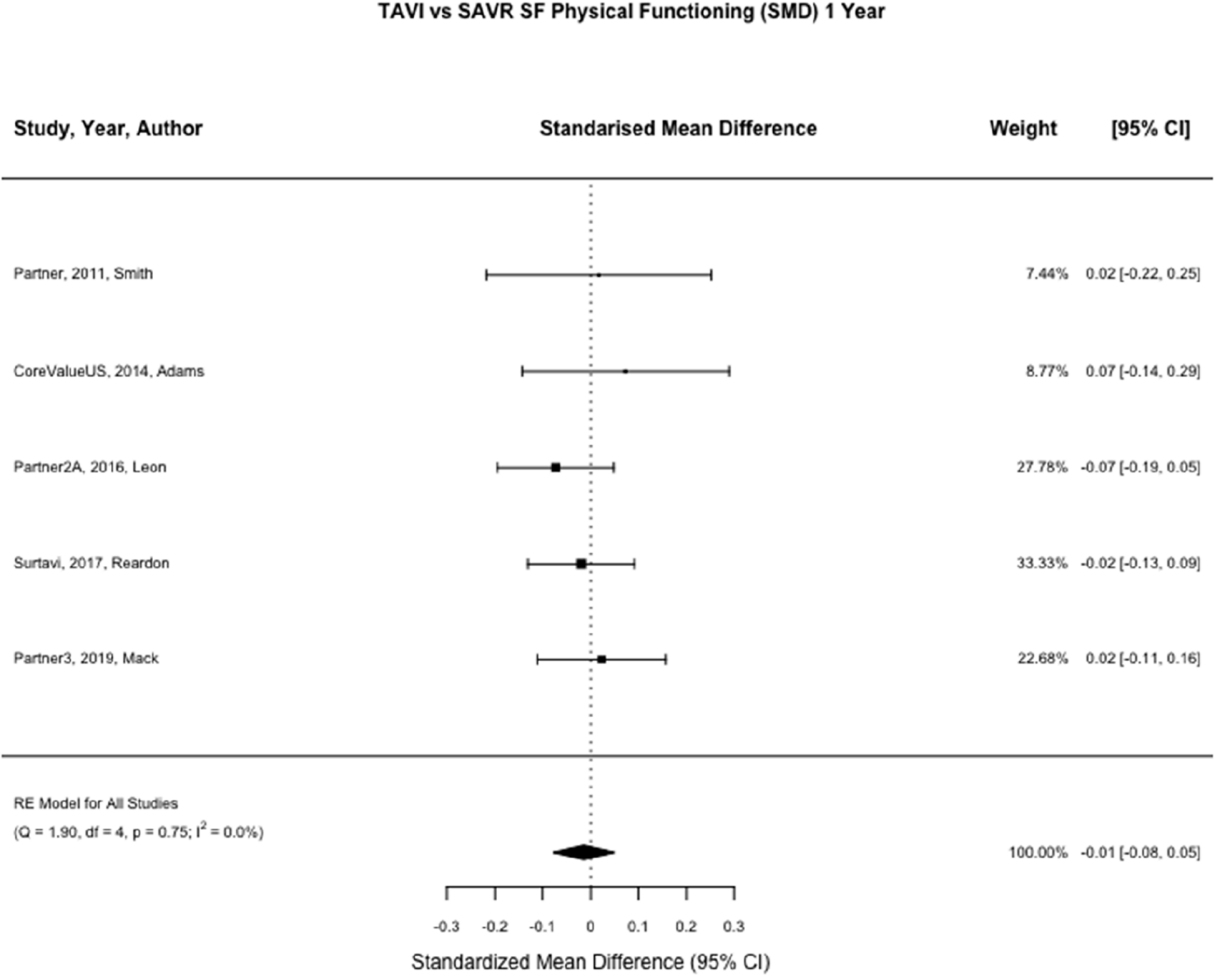
TAVI vs SAVR SF physical functioning (SMD) 1 year. Figure 9 shows a forest plot comparing the standardised mean difference of SF scores between TAVI and SAVR at one year. Abbreviations: SF Physical (Short Form Physical) TAVI (Transcatheter aortic valve implantation), SAVR (Surgical Aortic valve replacement), SMD (Standardised Mean Difference)

**Fig. 10:**
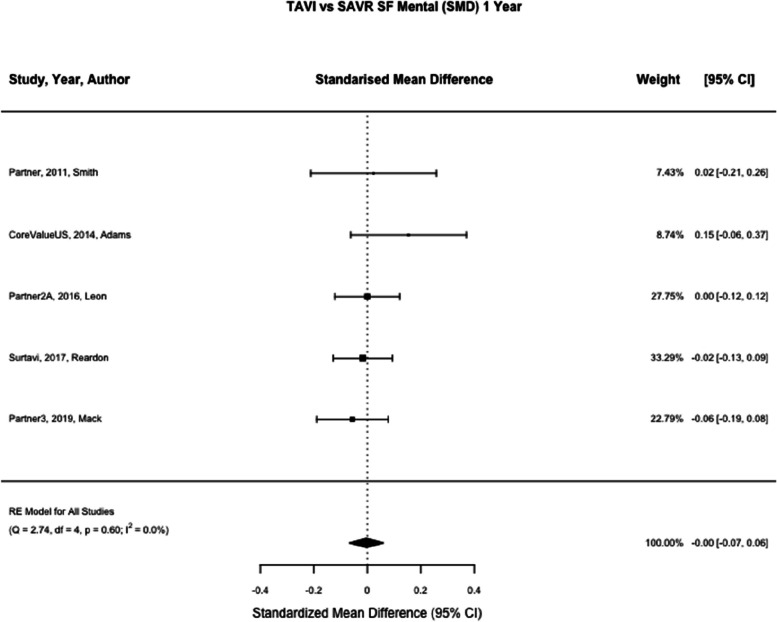
TAVI vs SAVR SF mental (SMD) 1 year. Figure 10 shows a forest plot comparing the standardised mean difference of SF scores between TAVI and SAVR at one year. Abbreviations: SF Mental (Short Form Mental) TAVI (Transcatheter aortic valve implantation), SAVR (Surgical Aortic valve replacement), SMD (Standardised Mean Difference)

SF-12 physical and mental summary scores was reported in two studies (Partner and CoreValueUS) [21, 22] and SF-36 physical and mental summary scores was reported in three studies (Partner2A, Surtavi and Partner3 [8, 23, 24] SF-12/36 was reported at one month in five studies for physical and mental summary scores (*n* = 4159, *n* = 4164) and one year in five studies for physical and mental summary scores (*n* = 3792, *n* = 3798) [8, 21–24]. At one month TAVI was associated with a greater standardised mean difference compared to SAVR, (SMD SF Physical Summary 0.55, 95% CI 0.31 – 0.78, *p* < 0.01, SMD SF Mental Summary 0.34, 95% CI 0.27 – 0.40, *p* < 0.01) (See Figs. 7 and 8). At one year there was no statistically significant difference between TAVI and SAVR (SMD SF Physical Summary -0.01, 95% CI -0.08 – 0.05, *p* = 0.67, SMD SF Mental Summary 0.00, 95% CI -0.07 – 0.06, *p* = 0.92) (Figs. 2, 9, and 10). Subgroup analysis by SF-12 and SF-36 scores did not materially alter the findings.

We conducted additional sensitivity analysis for the SF questionnaire to explore high heterogeneity at one month. Meta-regression analysis by year of study publication suggested that there was an association with more recent studies and an increased standardised mean difference favouring TAVI over SAVR (*p* = 0.003) at one month. This was not significant at one year. Meta-regression analysis by mean age of trial participants also suggested that there was an association with lower mean age and an increased standardised mean difference favouring TAVI over SAVR (*p* < 0.001) at one month. This was not significant at one year.

### NYHA

**Fig. 11:**
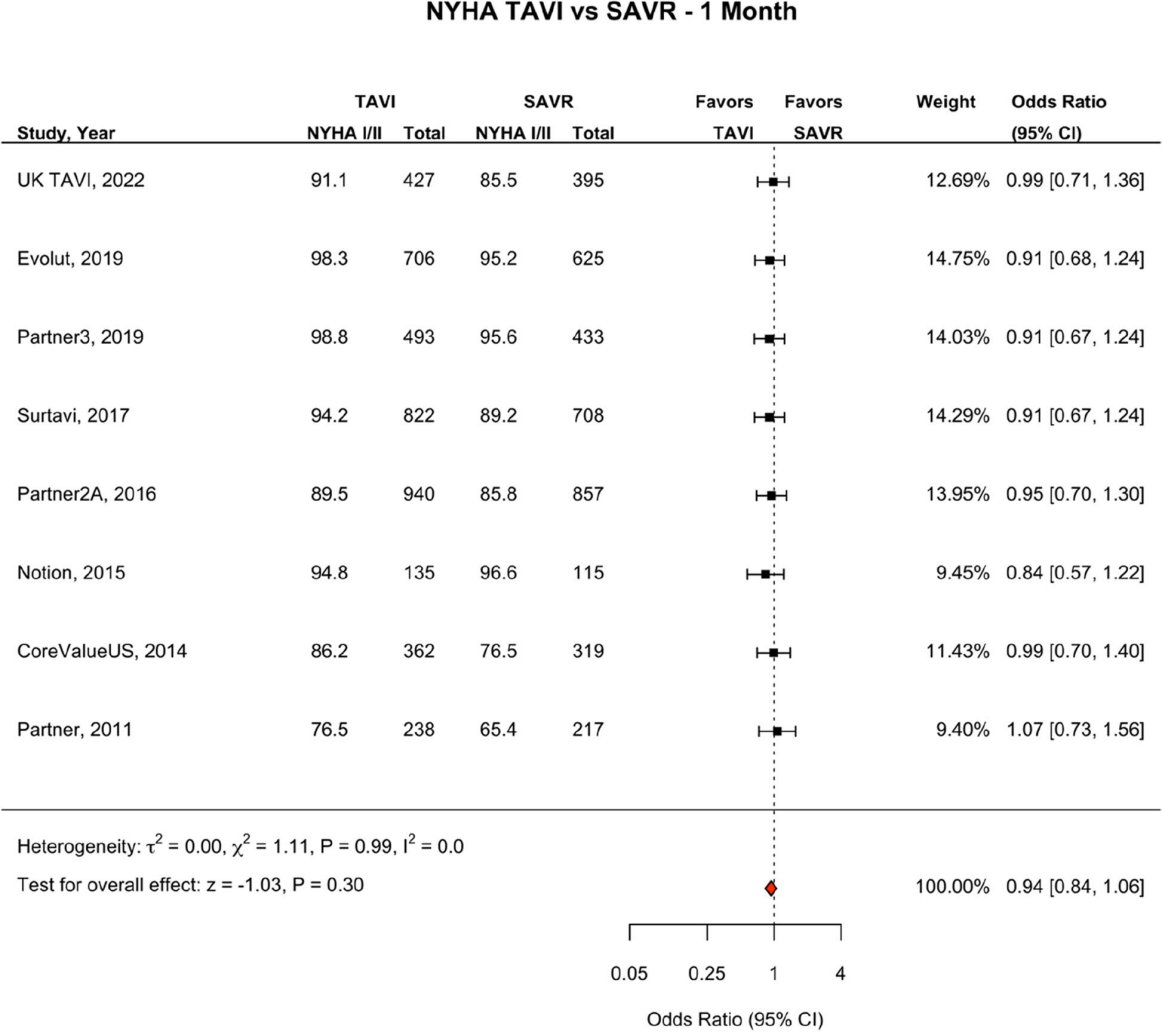
NYHA TAVI vs SAVR—1 month. Figure 11 shows a forest plot comparing odds of class III/IV NYHA status between TAVI vs SAVR at one month. Figure 11 shows a forest plot comparing odds of class III/IV NYHA status between TAVI vs SAVR at one year. Abbreviations: NYHA (New York Heart Association)

**Fig. 12:**
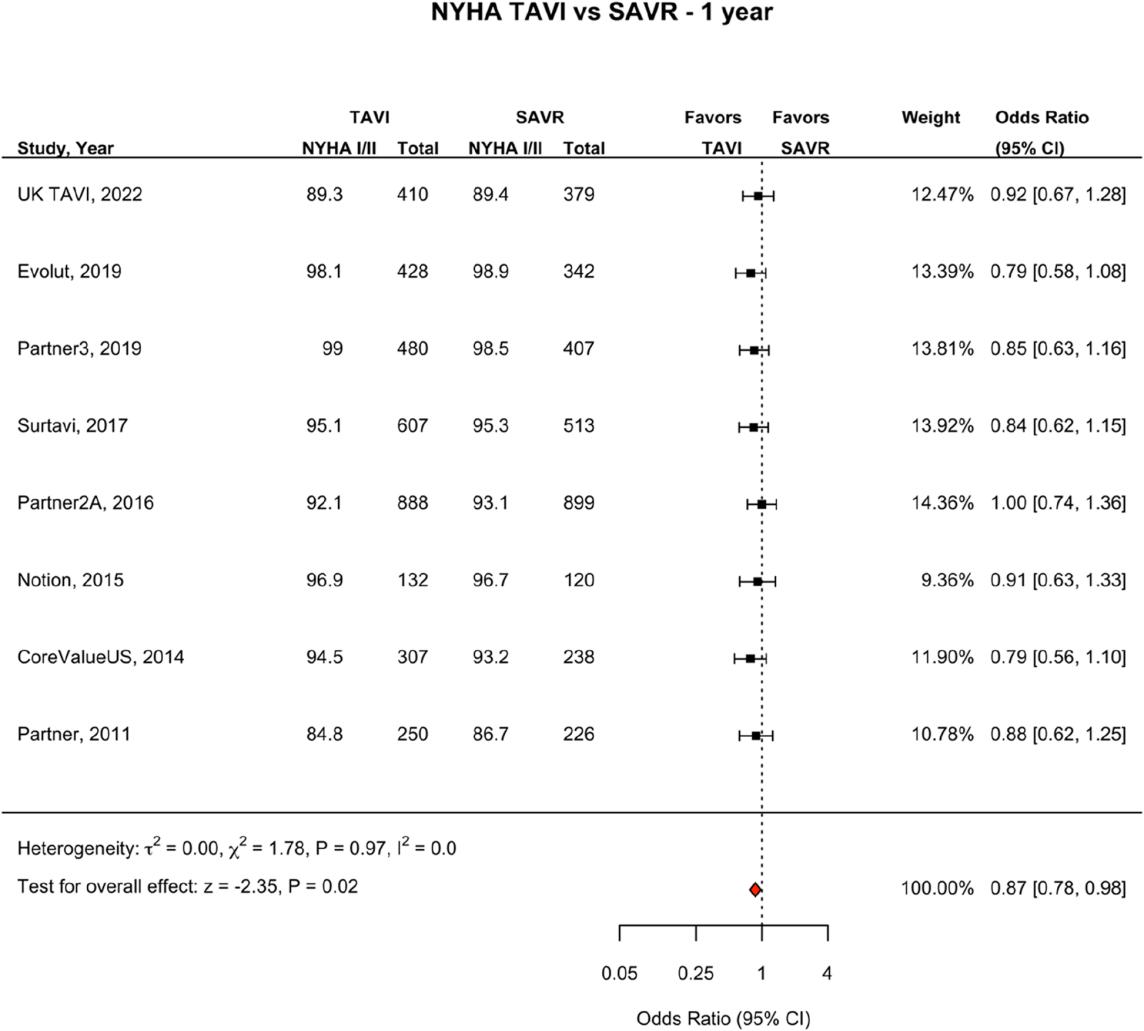
NYHA TAVI vs SAVR—1 year. Figure 12 shows a forest plot comparing the odds ratio of NYHA scores between TAVI and SAVR at one year. Abbreviations: NYHA (New York heart Association Score) TAVI (Transcatheter aortic valve implantation), SAVR (Surgical Aortic valve replacement), OR (Odds Ratio)

NYHA scale was reported at one month in eight studies (*n* = 7837, and one year in eight studies (*n* = 6626) [7–9, 20–24]. TAVI was not associated with reduced odds of NYHA class I/II compared to SAVR at one month (OR 0.94; 95% CI, 0.84–1.06, *p* = 0.30) (Fig. 11). TAVI was associated with a reduced odds of NYHA class I/II at one year (OR 0.87; 95% CI, 0.78–0.98, *p* = 0.02) (Fig. 12).

### Risk of bias

Risk of bias was assessed for 8 trials (Table 2). Each trial was graded as either “high risk”, “low risk” or “some concerns”. One trial was deemed ‘high risk’ for bias [21]. Some concern’ was found in the remaining 7 trials predominantly due to the risks associated with deviations from the intended interventions [7–9, 20–24]. Some concerns were also noted in the Partner 2A [23] trial due to differing rates of follow up between TAVI and SAVR at one month. The Notion trial [20] was found to have some concern regarding the measurement of outcomes due to the gathering of certain outcomes in an unblinded as to the procedure the participants underwent. Funnel plot evaluations did not reveal small study sample size bias (Supplementary Fig. 1).

**Table 2:** Risk of bias stratification for all included studies using the cochrane risk of bias 2 tool

Study	Overall Assessment	Domain 1: Randomisation	Domain 2: Bias due to deviations from the intended intervention	Domain 3: Bias due to missing outcome data	Domain 4: Bias in the measurement of the outcome	Domain 4: Bias in selection of the reported result
Partner: Risk of Bias	High	Low	High	Low	Low	Low
CoreValve US pivotal: Risk of Bias	Some Concerns	Low	Some Concerns	Low	Low	Low
Notion: Risk of Bias	Some Concerns	Low	Some Concerns	Low	Some Concerns	Low
Partner2A: Risk of Bias	Some Concerns	Low	Some Concerns	Some Concerns	Low	Low
Surtavi: Risk of Bias	Some Concerns	Low	Some Concerns	Low	Low	Low
Evolut: Risk of Bias	Some Concerns	Low	Some Concerns	Low	Low	Low
Partner3: Risk of Bias	Some Concerns	Low	Some Concerns	Low	Low	Low
UK TAVI: Risk of Bias	Some Concerns	Low	Some Concerns	Low	Low	Low

## Discussion

In this systematic review and meta-analysis, we found that both TAVI and SAVR were associated with improved quality of life scores. We found that short-term quality-of-life outcomes (one month post-procedure) favoured TAVI over SAVR for EQ5DL, KCCQ, and SF12/36 scores. However, there was no statistically significant difference in quality-of-life scores at one year. In contrast, there was no short-term difference in heart failure symptoms measured using NYHA score, however at one year TAVI was associated with reduced odds of NYHA class I/II (no limitation/slight limitation in physical activity).

### Improvements in quality-of-life scores across all patients

Patients who received either TAVI or SAVR had improved quality-of-life metrics at both time points examined in this systematic review. In addition, we found a consistent improvement in symptoms across the spectrum of high-risk patients to low-risk patients. This is an important finding highlighting that both patient groups benefit from a quality-of-life point of view irrespective of treatment received and that there are sustained early and late benefits.

### Comparison of quality of life scores in TAVI vs SAVR—EQ5DL and KCCQ

We found statistically significant improvements in both the KCCQ, SF 12/36 and EQ5DL summary scores at one month in favour of TAVI but no statistically significant difference in quality-of-life scores at one year. For KCCQ, there was no difference in any of the studies at one year. Potential reasons for this improvement in quality of life at one month could be attributed to less invasive techniques, lower levels of pain post-operatively, lower levels of anaesthesia used, lower rates of analgesia induced delirium, earlier mobilisation, earlier time to discharge, and lower rates of intra/postoperative complications [25–29]. Further benefits of TAVI over SAVR, such as less post-operative physical limitation and the potential for the next day's discharges, may be significant in shared decision-making between physician and patient [27]. The improvements in functional scores at one month have potential clinical implications when considering the overall impact on both patients and the healthcare providers.

The magnitude of the improvement in KCCQ identified here at one month also reflects an overall lower cost of treatment [30] meaning that the less invasive nature of TAVI when compared to SAVR may decrease the burden on carers, the cost of treatment and reduce the overall impact of treatment on patients’ lives.

EQ5DL was only reported in five of the eight RCTs, with data missing from two other low-risk patient populations. This is a significant limitation that we are potentially underpowered to identify if there may be a trend towards better functional outcomes in one year if more data is available for analysis. The only study that reported a difference in quality of life at one year was the UK TAVI trial which reported a difference in EQ5D5L at one year in favour of TAVI. The UK TAVI trial, the most recent large RCT published, was a pragmatic trial of patients across all surgical risk categories [9]. This may signal a trend towards improved quality of life scores at one year in the most contemporary study, and this is an important consideration when considering the real-world applicability of TAVI over SAVR.

At one year follow-up, no difference was observed between the groups regarding quality-of-life metrics. The lack of differences seen in global scores at one year is likely due to the resolution of early post-procedural complications. It is important to note that SAVR does not emerge as superior to TAVI at one year. This important aspect should be highlighted to pre-operative patients when deciding treatment options. The more significant burden of treatment and prolonged recovery period associated with SAVR have implications for length of hospitalisation and the potential for in-hospital muscular degeneration with negative consequences on discharge. The value of being at home to elderly populations has been repeatedly shown in the literature with impacts on self-perception of independence and should not be underestimated during joint decision making [31].

### Comparison of NYHA outcomes

In contrast to composite quality of life questionnaires, we found no significant difference in NYHA scores at one month but a difference in NYHA scores at one year, with patients undergoing a TAVI less likely to have an NYHA classification of 1 or 2. Due to variable reporting of the individual components of the NYHA, we dichotomised into a combined NHYA 1 or 2 classification which has the potential to introduce misclassification, meaning our findings must be interpreted with caution. There is also significant inter-observer variability in assessing this score, noted across the literature, meaning that our findings must be interpreted cautiously [32]. However, if this is a true finding, it may be of clinical significance as the subjective sensation of dyspnoea is a significant contributor to quality of life metrics and longer term follow up is needed.

### Future directions

In this systematic review and meta-analysis we have demonstrated that the benefit of TAVI over SAVR is preserved across all patient surgical risk groups. The indications for TAVI may be extended into areas such asymptomatic aortic stenosis, moderate aortic stenosis and severe aortic regurgitation and choice of a procedure that has a favourable impact on quality of life may influence shared decision-making conversations between physicians and patients [33]. Quality of life metrics should continue to be collected in ongoing studies of trans-cathether procedures and include both cardiac specific (e.g. KCCQ) and global metrics (SF12/36 and EQ5DL) to provide a comprehensive patient centred overview of the impact of these interventions. Older adults place importance on remaining at home which can preserve their sense of identity and self-independence [31] and choice of TAVI as a procedure would help to respect these wishes. Taken together these factors may influence shared decision-making conversations between physician and patient.

## Conclusion

In conclusion, TAVI has shown a statistically significant improvement in outcomes for quality of life 1-month post-operation compared to SAVR for EQ5DL, KCCQ and SF12/36, with no difference at one year. No significant change was observed for NYHA at one month. A significant difference was observed in favour of SAVR at one year for NYHA.

Association), TAVI (Transcatheter Aortic Valve Implantation), SAVR (Surgical Aortic Valve Replacement).

### Supplementary Information

**Additional file 1.**

## Data Availability

The datasets used and/or analysed during the current study are available from the corresponding author on reasonable request.

## References

[1] Commissioner O of the. FDA expands indication for several transcatheter heart valves to patients at low risk for death or major complications associated with open-heart surgery. FDA. 2020. https://www.fda.gov/news-events/press-announcements/fda-expands-indication-several-transcatheter-heart-valves-patients-low-risk-death-or-major. Accessed 8 Aug 2022.

[2] Synnott P, Murphy RP, Judge C, Costello M, Reddin C, Dennehy K (2021). Stroke Severity in Transcatheter Aortic Valve Implantation Versus Surgical Aortic Valve Replacement: A Systematic Review and Meta-Analysis. J Stroke Cerebrovasc Dis.

[3] Lazkani M, Singh N, Howe C, Patel N, Colón MJ, Tasset M (2019). An updated meta-analysis of TAVR in patients at intermediate risk for SAVR. Cardiovasc Revasc Med.

[4] Siontis GCM, Praz F, Pilgrim T, Mavridis D, Verma S, Salanti G (2016). Transcatheter aortic valve implantation vs. surgical aortic valve replacement for treatment of severe aortic stenosis: a meta-analysis of randomized trials. Eur Heart J.

[5] Vahanian A, Beyersdorf F, Praz F, Milojevic M, Baldus S, Bauersachs J (2022). 2021 ESC/EACTS Guidelines for the management of valvular heart disease: developed by the task force for the management of valvular heart disease of the European Society of Cardiology (ESC) and the European Association for Cardio-Thoracic Surgery (EACTS). Rev Esp Cardiol (Engl Ed).

[6] Ando T, Takagi H, Briasoulis A, Grines CL, Afonso L (2019). Comparison of health related quality of life in transcatheter versus surgical aortic valve replacement: a meta-analysis. Heart Lung Circ.

[7] Popma JJ, Deeb GM, Yakubov SJ, Mumtaz M, Gada H, O’Hair D (2019). Transcatheter aortic-valve replacement with a self-expanding valve in low-risk patients. N Engl J Med.

[8] Mack MJ, Leon MB, Thourani VH, Makkar R, Kodali SK, Russo M (2019). Transcatheter aortic-valve replacement with a balloon-expandable valve in low-risk patients. N Engl J Med.

[9] Toff WD, Hildick-Smith D, Kovac J, Mullen MJ, Wendler O, Mansouri A (2022). Effect of transcatheter aortic valve implantation vs surgical aortic valve replacement on all-cause mortality in patients with aortic stenosis: a randomized clinical trial. JAMA.

[10] Tuttle MK, Kiaii B, Van Mieghem NM, Laham RJ, Deeb GM, Windecker S (2022). Functional status after transcatheter and surgical aortic valve replacement: 2-year analysis from the SURTAVI trial. JACC Cardiovasc Interv.

[11] Forrest JK, Deeb GM, Yakubov SJ, Rovin JD, Mumtaz M, Gada H (2022). 2-Year outcomes after transcatheter versus surgical aortic valve replacement in low-risk patients. J Am Coll Cardiol.

[12] Higgins JPT. Cochrane Handbook for Systematic Reviews of Interventions version 6.0. Version 6.0. 2019. www.training.cochrane.org/handbook. Accessed 8 Jul 2022.

[13] Moher D, Liberati A, Tetzlaff J, Altman DG, Group P (2009). Preferred reporting items for systematic reviews and meta-analyses: The PRISMA statement. Ann Intern Med.

[14] Clarke M, Brice A, Chalmers I (2014). Accumulating research: a systematic account of how cumulative meta-analyses would have provided knowledge, improved health, reduced harm and saved resources. PLoS One.

[15] Ouzzani M, Hammady H, Fedorowicz Z, Elmagarmid A. Rayyan-a web and mobile app for systematic reviews. Syst Rev. 2016;5. 10.1186/s13643-016-0384-4 PMC5139140 27919275

[16] Ware J, Kosinski M, Keller S (1996). A 12-Item short-form health survey construction of scales and preliminary tests of reliability and validity. Med Care.

[17] Viechtbauer W (2010). Conducting Meta-Analyses in R with the metafor Package. J Stat Softw.

[18] Sterne JAC, Savović J, Page MJ, Elbers RG, Blencowe NS, Boutron I (2019). RoB 2: a revised tool for assessing risk of bias in randomised trials. BMJ.

[19] Nielsen HHM, Klaaborg KE, Nissen H, Terp K, Mortensen PE, Kjeldsen BJ (2012). A prospective, randomised trial of transapical transcatheter aortic valve implantation vs. surgical aortic valve replacement in operable elderly patients with aortic stenosis: The STACCATO trial. EuroIntervention.

[20] Gustav H, Thyregod H, Steinbrüchel DA, Ihlemann N, Nissen H, Kjeldsen BJ (2015). Transcatheter versus surgical aortic valve replacement in patients with severe aortic valve stenosis 1-year results from the all-comers NOTION randomized clinical trial. J Am Coll Cardiol.

[21] Smith CR, Leon MB, Mack MJ, Craig D, Moses JW, Svensson LG (2011). Transcatheter versus Surgical Aortic-Valve Replacement in High-Risk Patients. N Engl J Med.

[22] Adams DH, Popma JJ, Reardon MJ, Yakubov SJ, Coselli JS, Deeb GM (2014). Transcatheter aortic-valve replacement with a self-expanding prosthesis. N Engl J Med.

[23] Leon MB, Smith CR, Mack MJ, Makkar RR, Svensson LG, Kodali SK (2016). Transcatheter or surgical aortic-valve replacement in intermediate-risk patients. N Engl J Med.

[24] Reardon MJ, Van Mieghem NM, Popma JJ, Kleiman NS, Søndergaard L, Mumtaz M (2017). Surgical or transcatheter aortic-valve replacement in intermediate-risk patients. N Engl J Med.

[25] Toppen W, Johansen D, Sareh S, Fernandez J, Satou N, Patel KD (2017). Improved costs and outcomes with conscious sedation vs general anesthesia in TAVR patients: Time to wake up?. PLoS One.

[26] Shin SR, Kim WH, Kim DJ, Shin IW, Sohn JT (2016). Prediction and prevention of acute kidney injury after cardiac surgery. Biomed Res Int.

[27] Dolansky MA, Xu F, Zullo M, Shishehbor M, Moore SM, Rimm AA (2010). Post-acute care services received by older adults following a cardiac event: a population-based analysis. J Cardiovasc Nurs.

[28] Sirvinskas E, Andrejaitiene J, Raliene L, Nasvytis L, Karbonskiene A, Pilvinis V (2008). Cardiopulmonary bypass management and acute renal failure: risk factors and prognosis. Perfusion.

[29] Lauck SB, Yu M, Ding L, Hardiman S, Wong D, Sathananthan J (2021). Quality-of-life outcomes after transcatheter aortic valve implantation in a “Real World” population: insights from a prospective Canadian database. CJC Open.

[30] Chan PS, Soto G, Jones PG, Nallamothu BK, Zhang Z, Weintraub WS (2009). atient health status and costs in heart failure. Insights from the eplerenone post-acute myocardial infarction heart failure efficacy and survival study (EPHESUS). Circulation.

[31] Brown GC (2015). Living too long. EMBO Rep.

[32] Banovic M, Putnik S, Penicka M, Doros G, Deja MA, Kockova R (2022). Aortic valve replacement versus conservative treatment in asymptomatic severe aortic stenosis: The AVATAR trial. Circulation.

[33] Webb JG, Blanke P, Meier D, Sathananthan J, Lauck S, Chatfield AG (2022). TAVI in 2022: Remaining issues and future direction. Arch Cardiovasc Dis.

