# Supplementary material for: Comparing functional and quality of life outcomes in transcatheter aortic valve implantation and surgical aortic valve replacement for aortic stenosis: a systematic review and meta-analysis
Source: BMC Cardiovasc Disord. 2023 Oct 25;23:519. doi: 10.1186/s12872-023-03445-y (PMC10598915; doi:10.1186/s12872-023-03445-y)
Supplement: Supplementary file 1 — Additional file 1. [file 12872_2023_3445_MOESM1_ESM.docx]

|  | **KCCQ** | | | | | | | | | | | |
| --- | --- | --- | --- | --- | --- | --- | --- | --- | --- | --- | --- | --- |
|  | **One Month** | | | | | | **One Year** | | | | | |
| **Studies Used** | **nTAVI** | **Delta (CI 95%)** | **SD** | **nSAVR** | **Delta (C1 95%)** | **SD** | **nTAVI** | **Delta (CI 95%)** | **SD** | **nSAVR** | **Delta (CI 95%)** | **SD** |
| **Partner** | 197 | 23.70 (20.10, 27.30) | 25.80 | 157 | 12.10 (7.40, 16.70) | 29.70 | 165 | 24.4 (33.1, 28.5) | 28.5 | 136 | 26.8 (21.8, 31.7) | 29.4 |
| **Staccato** |  |  |  |  |  |  |  |  |  |  |  |  |
| **CoreValueUS** | 210 | 21.60 (17.70, 25.50) | 28.80 | 149 | 3.50 (-1.00, 7.90) | 27.70 | 205 | 24 (20.6, 27.5) | 25.2 | 163 | 21.8 (17.5, 26) | 27.7 |
| **Notion** |  |  |  |  |  |  |  |  |  |  |  |  |
| **Partner2A** | 678 | 17.50 (15.80, 19.30) | 23.20 | 551 | 3.20 (1.30, 5.50) | 25.10 | 596 | 22.1 (20.4, 23.9) | 21.8 | 479 | 22.1 (20.1, 24.1) | 22.3 |
| **Surtavi** | 819 | 18.40 | 22.76 | 700 | 5.88 | 27 | 607 | 20.9 | 22.3 | 513 | 20.6 | 22.3 |
| **Evolut** | 714 | 20 | 21.10 | 637 | 9.20 | 22.30 | 429 | 21.6 | 20.6 | 349 | 20.7 | 20.3 |
| **Partner3** | 490 | 18.50 (16.90, 20.10) | 24.90 | 429 | 2.50 (0.50, 4.60) | 33.60 | 479 | 19.4 (17.7, 21.2) | 24.3 | 400 | 17.4 (15.4, 19.3) | 27.4 |
| **UK TAVI** |  |  |  |  |  |  |  |  |  |  |  |  |

**Supplementary table 1. shows the data used in this study. nTAVI refers to the total number of TAVI patients included in this study. nSAVR refers to the total number of surgical patients included in this study. Delta (CI 95%) refers to the change from baseline with the 95% confidence interval included. SD refers to standard deviation. KCCQ refers to the Kansas City Cardiomyopathy Questionnaire.**

|  | **EQ5DL** | | | | | | | | | | | |
| --- | --- | --- | --- | --- | --- | --- | --- | --- | --- | --- | --- | --- |
|  | **One Month** | | | | | | **One Year** | | | | | |
| **Studies Used** | **nTAVI** | **Delta (CI 95%)** | **SD** | **nSAVR** | **Delta (C1 95%)** | **SD** | **nTAVI** | **Delta (CI 95%)** | **SD** | **nSAVR** | **Delta (CI 95%)** | **SD** |
| **Partner** | 192 | 0.08 (0.04, 0.11) | 0.25 | 154 | 0.02 (-0.02, 0.06) | 0.25 | 160 | 0.09 (0.05, 0.12) | 0.23 | 129 | 0.08 (0.04, 0.12) | 0.23 |
| **Staccato** |  |  |  |  |  |  |  |  |  |  |  |  |
| **CoreValueUS** | 204 | 0.055 (0.024, 0.087) | 0.23 | 144 | -0.073 (-0.116, -0.03) | 0.26 | 199 | 0.043 (0.015. 0.071) | 0.2 | 155 | 0.003 (-0.029, 0.035) | 0.2 |
| **Notion** |  |  |  |  |  |  |  |  |  |  |  |  |
| **Partner2A** | 675 | 0.058 (0.043, 0.072) | 0.19 | 543 | -0.002 (-0.019, 0.014) | 0.2 | 591 | 0.044 (0.029, 0.059) | 0.19 | 471 | 0.066 (0.048. 0.083) | 0.19 |
| **Surtavi** |  |  |  |  |  |  |  |  |  |  |  |  |
| **Evolut** |  |  |  |  |  |  |  |  |  |  |  |  |
| **Partner3** | 484 | 0.06 (0.05, 0.07) | 0.11 | 419 | -0.01 (-0.03, 0) | 0 | 475 | 0.04 (0.03, 0.05) | 0.11 | 391 | 0.04 (0.03, 0.06) | 0.15 |
| **UK TAVI** | 423 | 0.78 | 0.2 | 383 | 0.72 | 0.22 | 404 | 0.79 | 0.2 | 369 | 0.74 | 0.22 |

**Supplementary table 2 shows the data used in this study. nTAVI refers to the total number of TAVI patients included in this study. nSAVR refers to the total number of surgical patients included in this study. Delta (CI 95%) refers to the change from baseline with the 95% confidence interval included. SD refers to standard deviation. EQ5DL refers to European Quality of Life 5 Dimensions 5 Level Version**

|  | **SF Physical** | | | | | | | | | | | |
| --- | --- | --- | --- | --- | --- | --- | --- | --- | --- | --- | --- | --- |
|  | **One Month** | | | | | | **One Year** | | | | | |
| **Studies Used** | **nTAVI** | **Delta (CI 95%)** | **SD** | **nSAVR** | **Delta (C1 95%)** | **SD** | **nTAVI** | **Delta (CI 95%)** | **SD** | **nSAVR** | **Delta (CI 95%)** | **SD** |
| **Partner** | 184 | 5 (3.5, 6.4) | 10.03 | 149 | 2.6 (0.7, 4.4) | 11.52 | 155 | 6.3 (4.5, 8.2) |  | 127 | 6.1 (4.2, 8.1) |  |
| **Staccato** |  |  |  |  |  |  |  |  |  |  |  |  |
| **CoreValueUS** | 186 | 5.4 (4, 6.9) | 10.08 | 137 | 0 (-1.7, 1.7) | 10.15 | 187 | 5.9 (4.2, 7.5) |  | 147 | 5.1 (3.4, 6.7) |  |
| **Notion** |  |  |  |  |  |  |  |  |  |  |  |  |
| **Partner2A** | 669 | 4.6 (3.9, 5.3) | 9.23 | 532 | 1 (-0.8, 0.8) | 9.41 | 585 | 4.4 (3.7, 5.2) |  | 470 | 5.1 (4.2, 6) |  |
| **Surtavi** | 770 | 5.8 (5, 6.5) | 10.61 | 637 | -1 (-1.8, -0.1) | 10.94 | 695 | 5.2 (4.4, 5.9) |  | 568 | 5.4 (4.5, 6.2) |  |
| **Evolut** |  |  |  |  |  |  |  |  |  |  |  |  |
| **Partner3** | 479 | 5 (4.3, 5.7) | 7.81 | 416 | -2.7 (-3.6, -1.9) | 8.84 | 469 | 5.2 (4.4, 6) |  | 389 | 5 (4.2, 5.9) |  |
| **UK TAVI** |  |  |  |  |  |  |  |  |  |  |  |  |

**Supplementary table 3 shows the data used in this study. nTAVI refers to the total number of TAVI patients included in this study. nSAVR refers to the total number of surgical patients included in this study. Delta (CI 95%) refers to the change from baseline with the 95% confidence interval included. SD refers to standard deviation. SF refers to the short form questionnaire.**

|  | **SF Mental** | | | | | | | | | | | |
| --- | --- | --- | --- | --- | --- | --- | --- | --- | --- | --- | --- | --- |
|  | **One Month** | | | | | | **One Year** | | | | | |
| **Studies Used** | **nTAVI** | **Delta (CI 95%)** | **SD** | **nSAVR** | **Delta (C1 95%)** | **SD** | **nTAVI** | **Delta (CI 95%)** | **SD** | **nSAVR** | **Delta (CI 95%)** | **SD** |
| **Partner** | 184 | 4.3 (2.5, 6.1) |  | 149 | -0.3 (-2.6, 2.1) |  | 155 | 5 (3.1, 7) |  | 127 | 4.7 (2.4, 6.9) |  |
| **Staccato** |  |  |  |  |  |  |  |  |  |  |  |  |
| **CoreValueUS** | 186 | 3.5 (1.7, 5.4) |  | 137 | -2.9 (-5.1, -0.7) |  | 187 | 4.8 (3, 6.5) |  | 147 | 2.9 (0.9, 4.9) |  |
| **Notion** |  |  |  |  |  |  |  |  |  |  |  |  |
| **Partner2A** | 669 | 2.4 (1.5, 3.4) |  | 532 | -2.6 (-3.8, -1.4) |  | 532 | -2.6 (-3.8, -1.4) |  | 585 | 3.3 (2.3, 4.2) |  |
| **Surtavi** | 770 | 2.9 (2.1, 3.8) |  | 637 | -0.4 (-1.4, 0.7) |  | 695 | 4 (3.2, 4.9) |  | 568 | 4.2 (3.2, 5.2) |  |
| **Evolut** |  |  |  |  |  |  |  |  |  |  |  |  |
| **Partner3** | 483 | 3.4 (2.6, 4.2) |  | 417 | 0.1 (-1, 1.1) |  | 473 | 3.5 (2.7, 4.3) |  | 391 | 4 (3.1, 4.9) |  |
| **UK TAVI** |  |  |  |  |  |  |  |  |  |  |  |  |

**Supplementary table 4 shows the data used in this study. nTAVI refers to the total number of TAVI patients included in this study. nSAVR refers to the total number of surgical patients included in this study. Delta (CI 95%) refers to the change from baseline with the 95% confidence interval included. SD refers to standard deviation. SF refers to the short form questionnaire.**

**Supplementary Figure 1: KCCQ Funnel Plot**

**
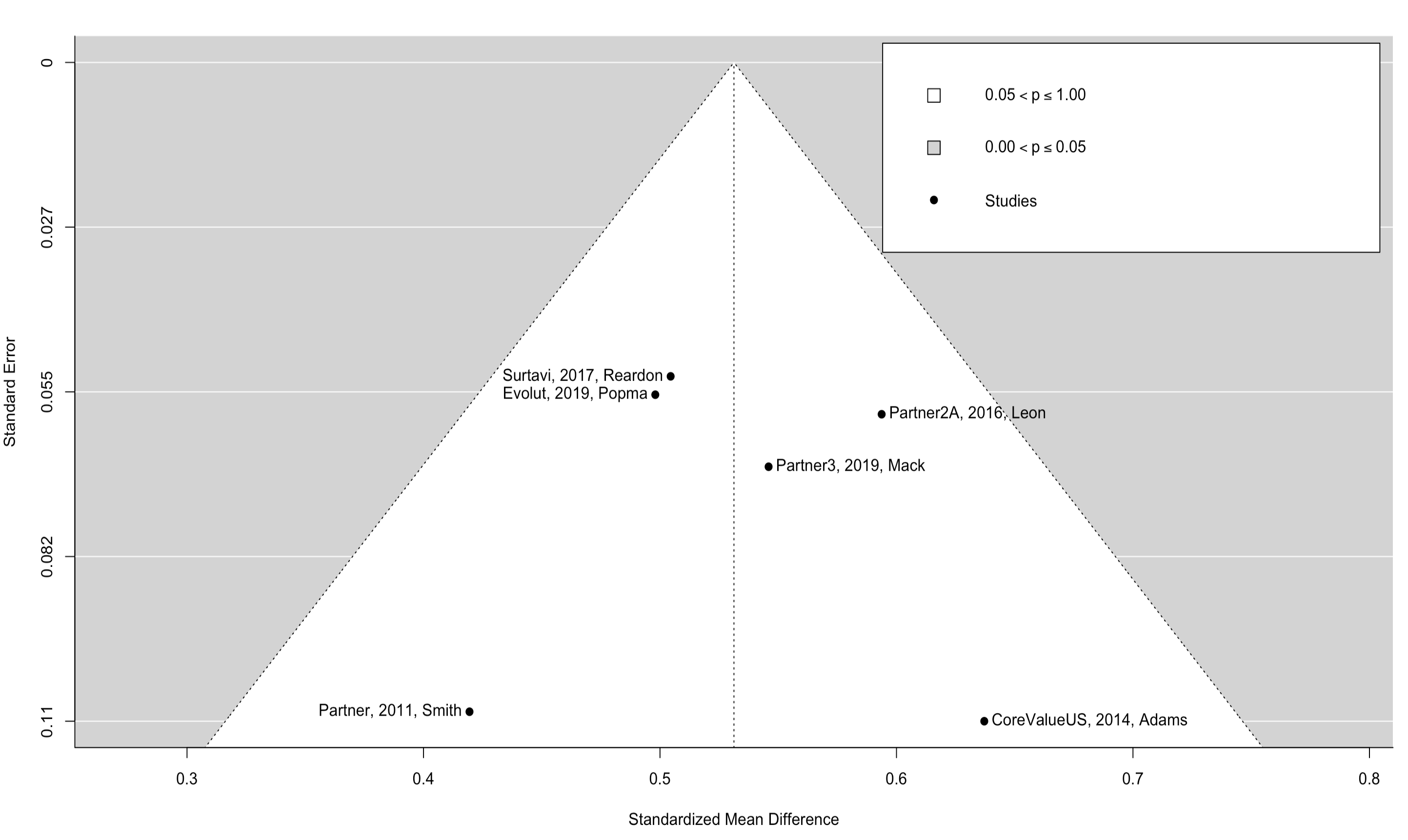
**

**Supplementary Figure 2: EQ5DL Funnel Plot**

**
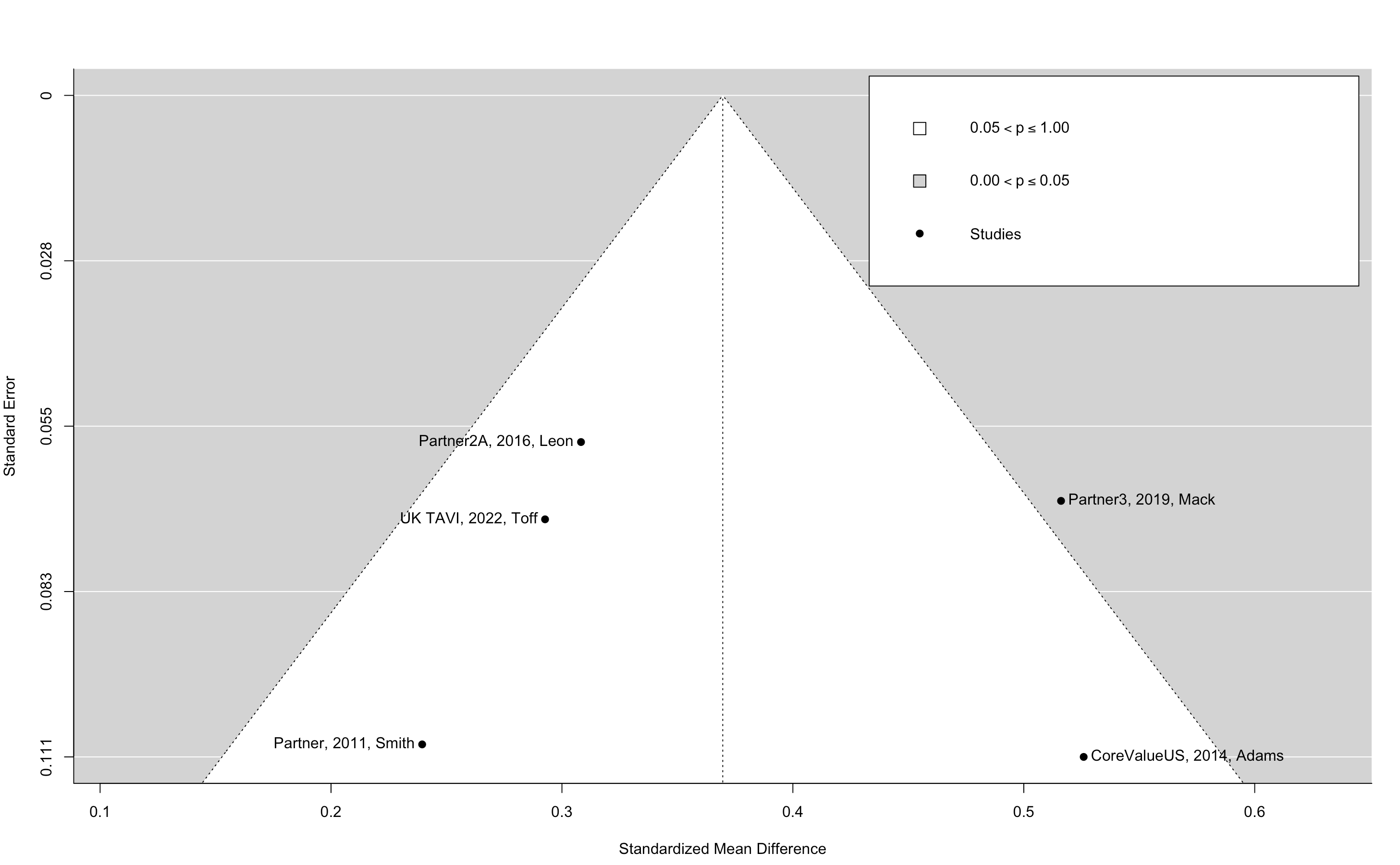
**

**Supplementary Figure 3: SF 1 month Physical Summary**

**
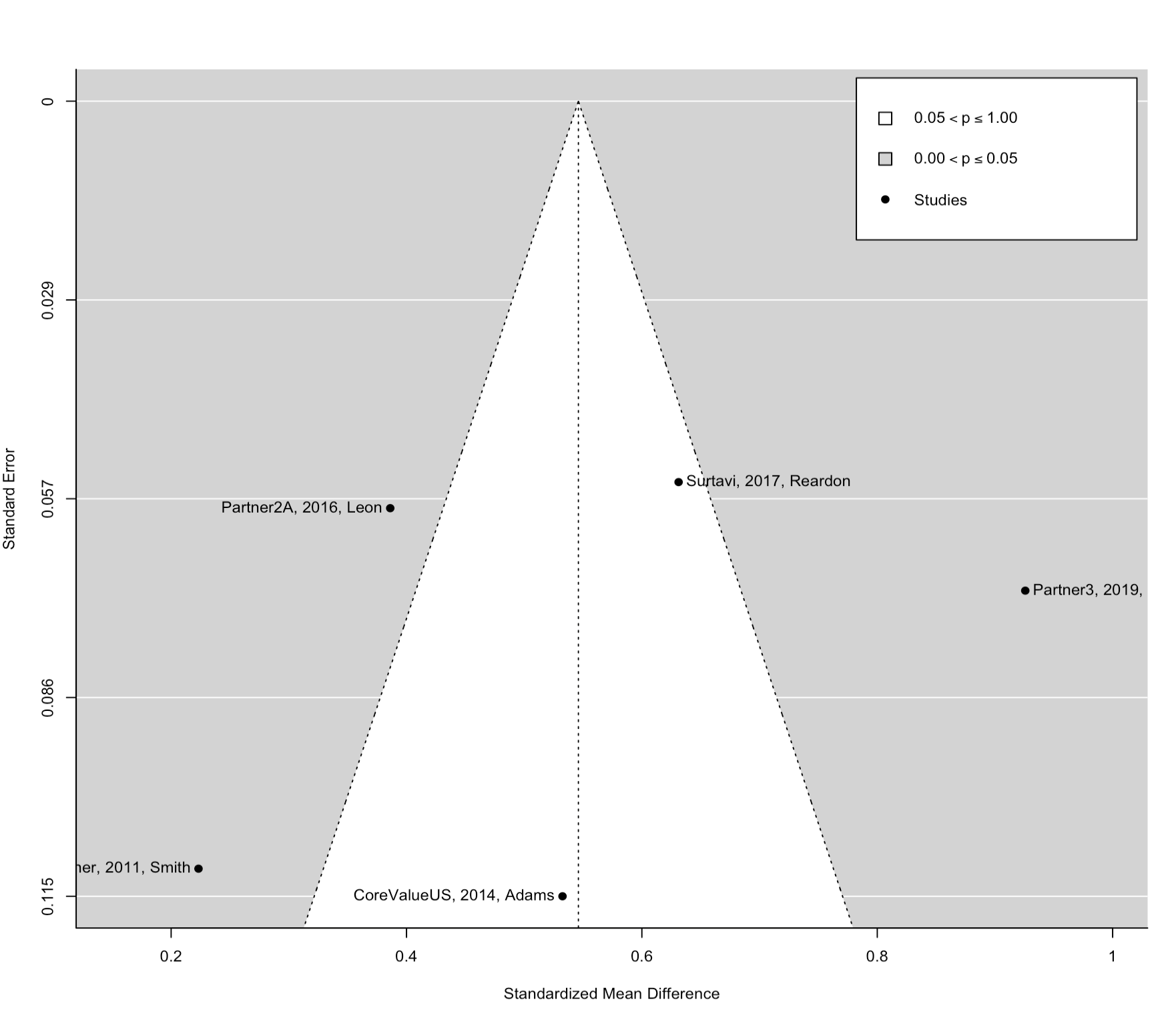
**
